# Prevalence of Malnutrition and Associated Factors among Children in Rural Ethiopia

**DOI:** 10.1155/2017/6587853

**Published:** 2017-05-17

**Authors:** Neima Endris, Henok Asefa, Lamessa Dube

**Affiliations:** Department of Epidemiology, Jimma University, Jimma, Ethiopia

## Abstract

**Background:**

Child malnutrition continues to be the leading public health problem in developing countries. In Ethiopia, malnutrition is a leading cause of child illness and death. Recently the composite index of anthropometric failure (CIAF) has been implemented to measure the prevalence of malnutrition. This index presents a more complete picture compared with the previous conventional indices. In this study, CIAF was used to determine the prevalence of malnutrition among children aged 0–59 months in rural Ethiopia.

**Methods:**

Data was extracted from the 2014 Ethiopian Mini Demographic and Health Survey (EMDHS) for this study. A total of 3095 children were included in the analysis. The composite index of anthropometric failure (CIAF) was used to measure the nutritional status of the children. Logistic regression was fitted, to identify factors associated with malnutrition among children in rural Ethiopia, using STATA 13.

**Result:**

The prevalence of malnutrition among rural children in Ethiopia was 48.5%. Age of the children, preceding birth interval, educated status of mother, wealth status, and region were factors independently associated with nutritional status of children in rural Ethiopia.

**Conclusion:**

The prevalence of malnutrition among children in rural Ethiopia was high. A child older than 12 months, having uneducated mother, living in a household with poor wealth status, born with short birth interval, and living in some region of the country are associated with increased odds of being malnourished.

## 1. Introduction

Child malnutrition continues to be the leading public health problem in developing countries. Globally, there were 165 million stunted, 99 million underweight, and 51 million wasting children by year 2012. It kills 3.1 million under-five children every year [1]. Under-five children are the most susceptible age group for malnutrition. Malnutrition at the early stages of life can increase risk infections, morbidity, and mortality together with decreased mental and cognitive development. The effect of child malnutrition is long lasting and goes beyond childhood. For instance, malnutrition during early age decreases the educational achievement and labor productivity and raises the risk of chronic illnesses in later age [1–3].

Malnutrition is the major cause of illness and death among under-five children in Ethiopia. The rate of malnutrition among under-five children in the country is among the highest in the world and Sub-Saharan Africa. Moreover, malnutrition is the underlying cause for three-fifth of child death in the country [4–6]. According to the 2014 Ethiopian Mini Demographic and Health Survey (EMDHS) report, 42%, 26.7%, and 9% of under-five children were stunted, underweight, and wasted, respectively. The problem is even worse in rural areas. For instance, the prevalence of underweight and stunting among rural children was 27% and 42% compared with only 13% and 24% among urban children, respectively [7].

The planning of an appropriate intervention requires the knowledge of the extent and the underlying causes of the problem. To this end, very few studies have been conducted regarding childhood malnutrition in rural Ethiopia. All of them were small scale surveys limited in particular regions of the country [4, 6, 8–10]. Hence, they did not provide a full picture of the extent of the problem on country level. Moreover, these studies used the conventional indicators of nutritional status to measure the prevalence of malnutrition in under-five children. However, a number of studies pointed out that the use of conventional indicators provides only the categorization of children into the general categories of malnutrition and does not determine the overall prevalence of malnutrition associated with multiple failures. Consequently, these indicators underestimate the prevalence of malnutrition due to the potential overlap of children into multiple categories of anthropometric failure [11–16].

Therefore, in this study we used a recently developed and relatively robust alternative indicator of malnutrition—the composite index of anthropometric failure (CIAF) to determine the prevalence of malnutrition and associated factors among under-five children in rural Ethiopia.

## 2. Methods

### 2.1. Data Source

For this study, we used the 2014 Mini Ethiopian Demographic and Health Survey data. A nationwide survey was conducted by the Ethiopian Central Statistical Agency (ECSA) under the aegis of the Ministry of Health for four months from January to April 2014. A total of 8,475 households 2,556 from urban and 5,919 from rural areas were covered. A two-stage stratified-cluster sampling design was used to select these households [7].

Then data was extracted, on children aged 0–59 months, from this survey data to determine the prevalence of malnutrition and the associated factors among rural children in Ethiopia using CIAF as a measure of nutritional status of the children. In household where there were more than one under-five child, data of only the younger one was extracted.

### 2.2. Measurements

The outcome variable of interest in this study was nutritional status among under-five children measured using CIAF. Children are categorized into seven groups, namely, no failure; wasting only; wasting and underweight; wasting, stunting, and underweight; stunting and underweight; stunting only; and underweight only. A child who did not suffer from any anthropometric failure was classified as “no failure.” A child with acceptable Weight-for-Age (WA) and Height-for-Age (HA) but who had subnormal Weight-for-Height (WH) (that is, below −2 z-scores of the WHO 2006 standards) was classified as “wasting only.” A child with acceptable WA and HA but who had subnormal WH (that is, below −2 z-scores of the WHO 2006 standards) was classified as “wasting only.” A child with acceptable HA but low WA and WH (that is, below −2 z-scores of the WHO 2006 standards) was classified “wasting and underweight.” A child who suffered from anthropometric failure on all three measures was “wasting, stunting and underweight.” A child with low WA and HA (that is, below −2 z-scores of the WHO 2006 standards) but acceptable WH was classified as “stunting and underweight.” A child with low HA (that is, below −2 z-scores of the WHO 2006 standards) but acceptable WA and WH was categorized as “stunting only.” A child with low WA only (that is, below −2 z-scores of the WHO 2006 standards) was categorized as “underweight only.” Thus a child is regarded as malnourished, as measured in CIAF, if s/he is suffering from any anthropometric failure above [11, 14, 15].

A range of information was extracted from the Mini EDHS including (i) sociodemographic and economic characteristics (child's age, child's sex, birth order, preceding birth interval, ANC visit, maternal marital status, maternal educational status, maternal age at first birth, size of household, head of household, region of residence, and wealth index). The preceding birth interval was computed from the data collected by questions “Now I would like to record birth date of all your births, whether still alive or not, starting with the first one you had.” Except for the first born child, the preceding birth interval was computed by calculating the deference between the last and the preceding birth dates. The current marital status was categorized into currently in union or not in union. Legal or formal married women and an informal union in which a man and a woman live together were categorized as currently in union, otherwise not in union. ANC visit was measured by directing “For last birth, did you see anyone for antenatal care for during pregnancy?” a yes or no question.

The household wealth index is a socioeconomic index constructed as an indicator of the level of wealth that is consistent with expenditure and income measures. In the EDHS the index is based on data from household ownership of assets and consumer goods such as source of drinking water, type of toilet facilities, type of fuel, ownership of various durable goods, and other characteristics relating to socioeconomic status of the household. A factor score generated through principal components analysis was assigned to each asset, and the resulting asset scores were standardized in relation to a normal distribution [7]. For this analysis, the wealth index was grouped into three categories: poor, middle, and rich.

### 2.3. Data Analysis

Data were extracted from EDHS 2014 database, exported, edited, cleaned, coded, and analyzed using STATA version 13 for Windows. Then a weighted analysis was conducted using the same sampling weight given for each region by EDHS to make the data nationally representative [7]. First, descriptive analysis was done. Then bivariate analysis was done to identify factors associated with malnutrition. Those variables in the bivariate analysis with *P* value less than 0.25 were considered as candidates to be included in the multivariable logistic regression model. The multivariable logistic regression was performed by the backward stepwise variable selection method with probability of removal 0.10. Finally, the adequacy of the model was checked by using Hosmer and Lemeshow goodness-of-fit test.

## 3. Results

### 3.1. Socioeconomic and Demographic Characteristics of Study Participants

A total of 3095 weighted children living in rural area were included in the analysis among which 50.1% were females. The mean age of the children was 26.4 ± 16.2 months. Mean age of the mother at first birth was 18.7 ± 3.4 years. Majority of mothers (93%) were in union by marital status. Almost 87% of the children had preceding birth interval greater than twelve months. More than half (56.5%) of the children were living in a household with more than five members. Majority of the mothers (67.3%) had no education. Most of the households (89.8%) were male headed and 50.5% were found in the poor wealth quintile (Table 1).

### 3.2. Prevalence of Malnutrition

Based on CIAF, 48.5% of children living in rural Ethiopia were malnourished. From this, 22.7% had single anthropometric failure and 25.9% had multiple anthropometric failures. The prevalences of the seven groups of nutritional status were presented in Table 2. Malnutrition among rural children in Ethiopia using the conventional measures was found to be underweight 27%, wasting 9.7%, and stunting 41.2%.

### 3.3. Factors Associated with Nutritional Status of Children

The result of multivariable logistic regression analysis showed that age of the child, preceding birth interval, region of residence, maternal education, and economic status of the household were factors independently associated with nutritional status of children.

Compared to children less than six months old, the odds of malnutrition among children in the age group of 12–23 months were 2.6 times higher [AOR = 2.63, 95% CI: 1.72–4.01], in the age group of 24–35 months were 4 times higher [AOR = 3.97, 95% CI: 2.52–6.26], in the age group of 36–47 months were 3.5 times higher [AOR = 3.51, 95% CI: 2.32–5.31], and in the age group of 48–59 months were 2.75 times higher [AOR = 2.75, 95% CI: 1.64–4.63].

Children whose preceding birth interval was less than two years were 1.43 times at higher risk of being malnourished compared to children with preceding birth interval greater than 24 months [adjusted odds ratio (AOR) = 1.43, 95% CI: 1.02–2.04]. The risk of being malnourished among children whose mother did not attend education was 1.32 times higher compared to children whose mothers attended primary education [AOR = 1.32, 95% CI: 1.02–1.72].

Compared to children residing in households with poor economic status, the probability of being malnutrition among children residing in households with medium and rich economic status decreased by 34% and 37%, respectively. This study also showed that when compared to children in Tigray region, the risk of malnutrition was decreased by 55% [AOR = 0.47, 95% CI: 0.26–0.82], 41% [AOR = 0.59, 95% CI: 0.37–0.98], and 41% [AOR = 0.59, 95% CI: 0.36–0.97] among children living in Gambela, Southern Nations Nationalities and Peoples (SNNP), and Harari regions, respectively (Table 3).

## 4. Discussion

The findings of this study revealed that almost half of children aged 0–59 months were malnourished and the risk factors were region of residence, education of mother, economic status, age of child, and preceding birth interval.

The prevalence of malnutrition among rural children was 48.5% based on CIAF measurement. This prevalence is higher compared with the results of the Ethiopian Demographic and Health Survey and regional as well as national levels studies [4, 7, 17]. This difference might be due to the difference in the methods used to assess nutritional status. A number of literatures reported that the conventional methods used to assess malnutrition in a population underestimate the prevalence of malnutrition compared with CIAF [14, 15]. Estimated CIAF of African countries based on 2008 data ranges from 35.6% in Ghana to 51.6% [16]. Compared to Asian countries like China and India where reported CIAF are 21.7% and 32.7%, respectively [14, 15], the result of this study is higher.

In this study, age of the child was found to be significantly associated with nutritional status, as the age of child increases the risk of being malnourished increases. This finding is in line with studies done in Tigray region, Nigeria, and Bangladesh [6, 18, 19]. One conceivable clarification could be because of the late introduction of supplementary food with low nutritional quality [14]. The other reason might be that a large portion of guardians in rural areas are neglecting to fulfill optimal food requirements of their children as the child's age increases [20].

Preceding birth interval is the other important variable which is associated with nutritional status of children. In particular, there is an inverse relationship between the length of the preceding birth interval and the proportion of children who are malnourished. This finding is also consistent with the report of Ethiopian DHS 2011 and 2014 and other studies conducted in Ethiopia, Nigeria, India, and Bangladesh [4, 7, 18–21]. For the newborns, the larger birth interval results into better care and more time allocation for the nutrition and wellbeing [20].

This study demonstrated that there is significant association between maternal education and nutritional status of children. This finding is consistent with other studies done in Ethiopia, Bangladesh, and Nigeria [8, 14, 18]. This is because educated mothers are more conscious about their children's health; and they tend to look after their children in a better way [18].

This study also indicated that there is association between region of residence and nutritional status. Earlier survey has also shown a very low prevalence of malnutrition in these regions [22]. The watched distinction may reflect natural imperatives, more awful broad living conditions, harmful sociocultural practices, unequal intrahousehold food distribution, seasonal food insecurity, poor public facilities, and other related factors [21].

In addition, our study showed that under-five children from poor wealth status are at a higher risk of malnutrition compared with children from rich households. This is consistent with other studies done in Ethiopia, Bangladesh, Nigeria, and India [4, 14, 18, 23].

Finally, this study is limited in that it does not take into account some important variables that affect the nutritional status of children such as dietary aspects.

## 5. Conclusions

In conclusion, prevalence of malnutrition among under-five children in rural Ethiopia was high. Malnutrition continued to be a substantial burden in the country. And age of the child, maternal education, wealth status, and birth interval were associated with nutritional status of children in rural Ethiopia.

Special attention needs to be given to the problem of malnutrition among rural children in the country since the problem is extensive compared to previous studies done using the conventional methods of assessing nutritional status. We recommend policy makers to use CIAF as a measure of nutritional status in order to estimate the overall burden of malnutrition in the population.

## Figures and Tables

**Table 1:** Demographic and socioeconomic characteristics of children aged 0–59 months, rural Ethiopia, 2014.

Characteristics	Frequency (%)
*Sex of the child*	
Male	1545 (49.9)
Female	1550 (50.1)

*Age of child (in months)*	
<6	346.6 (11.2)
6–11	356.3 (11.5)
12–23	694.1 (22.4)
24–35	687.9 (22.2)
36–47	592.1 (19.1)
48–59	417.7 (13.5)

*Birth order*	
1	405.8 (14.4)
2-3	862.1 (30.6)
4-5	758.9 (27.0)
6^+^	793.5 (28.1)

*Preceding birth interval*	
≤24 months	406 (13.1)
>24 months	2689 (86.9)

*Current marital status*	
Currently not in union	116 (7.0)
Currently in union	1550 (93.0)

*Mother age at first birth *	
18 and less	917.2 (55.6)
19 and above	734 (44.4)

*Household family size*	
Five and less	1346 (43.5)
Six and above	1749 (56.5)

*Region of residence*	
Oromiya	1264 (40.9)
Amhara	754.6 (24.4)
SNNP	741.3 (23.9)
Tigray	198 (6.4)
Somalia	55.1 (1.8)
Benshangul-Gumuz	31.2 (1.0)
Affar	26.2 (0.8)
Gambela	12.4 (0.4)
Dire Dawa	7.2 (0.2)
Harari	4.6 (0.15)

*Mother's education*	
No education	1898 (67.3)
Primary education	848.8 (30.1)
Secondary and above	73.2 (2.6)

*Sex of household head*	
Male	2778 (89.8)
Female	316.3 (10.2)
Two and less	2797 (90.4)
More than two	298 (9.6)

*Wealth status*	
Poor	1562 (50.5)
Medium	717.4 (23.2)
Rich	815.2 (26.3)

*ANC visit *	
Yes	1250 (55.7)
No	994 (44.3)

**Table 2:** Prevalence of malnutrition based on CIAF among under-five-year-old children in rural Ethiopia, 2014.

Group	Description of the group	Frequency (percentage)
I	No failure	1594 (51.5%)
II	Wasting only	101.4 (3.3%)
III	Wasting and underweight	85.3 (2.8%)
IV	Wasting, stunting, and underweight	113 (3.7%)
V	Stunting and underweight	599.2 (19.4%)
VI	Stunting only	563 (18.2%)
VII	Underweight only	39 (1.3%)

Total		3094.9 (100%)

CIAF (II + III + IV + V + VI + VII) = *48.5%*.

**Table 3:** Factors independently associated with nutritional status of rural children in Ethiopia, 2014.

Variables	Adjusted OR	95% CI
*Age of child (in months)*		
<6	1	
6–11	0.78	0.48–1.24
12–23	2.63	1.72–4.01^*∗*^
24–35	3.97	2.52–6.26^*∗*^
36–47	3.51	2.32–5.31^*∗*^
48–59	2.75	1.64–4.63^*∗*^

*Preceding birth interval*		
>24 months	1	
≤24 months	1.43	1.023–2.04^*∗*^

*Mother's educational status*		
Primary	1	
No education	1.32	1.02–1.72^*∗*^
Secondary and above	0.49	0.19–1.29

*Region of residence*		
Tigray	1	
Afar	1.12	0.62–2.06
Amhara	0.71	0.44–1.15
Oromiya	0.62	0.38–1.00
Somali	0.79	0.46–1.37
Benshangul-Gumuz	0.70	0.37–1.32
SNNP	0.59	0.37–0.98^*∗*^
Gambella	0.47	0.26–0.82^*∗*^
Harari	0.59	0.36–0.97^*∗*^
Dire Dawa	0.78	0.43–1.42

*Wealth status*		
Poor	1	
Medium	0.66	0.45–0.95^*∗*^
Rich	0.63	0.62–0.65^*∗*^

*Note*. ^*∗*^Significant at *P* value of 0.05; SNNP: Southern Nations Nationalities and Peoples.

## Abbreviations

ANC: Antenatal care
AOR: Adjusted odds ratio
CIAF: Composite index of anthropometric failure
CSA: Central Statistical Agency
EMDHS: Ethiopian Mini Demographic and Health Survey
SNNPS: South Nations Nationalities and Peoples Regional State.

## Additional Points

*Availability of Data and Material.* The dataset supporting the conclusions of this study is not available because this study is based on national data and we have no mandate to share the dataset.
